# In Vitro Modulation of Complement Activation by Therapeutically Prospective Analogues of the Marine Polychaeta Arenicin Peptides

**DOI:** 10.3390/md20100612

**Published:** 2022-09-28

**Authors:** Ilia A. Krenev, Pavel V. Panteleev, Ekaterina S. Umnyakova, Nikolay P. Gorbunov, Valeria A. Kostevich, Sergey V. Balandin, Tatiana V. Ovchinnikova, Galina M. Aleshina, Mikhail N. Berlov

**Affiliations:** 1 Department of General Pathology and Pathological Physiology, Institute of Experimental Medicine, Academician Pavlov Street 12, 197376 Saint Petersburg, Russia; 2 M.M. Shemyakin and Yu. A. Ovchinnikov Institute of Bioorganic Chemistry, Russian Academy of Sciences, Miklukho-Maklaya Street, 16/10, 117997 Moscow, Russia; 3 Department of Pharmaceutical Sciences, University of Basel, Klingelbergstrasse 50, 4056 Basel, Switzerland; 4 Department of Molecular Genetics, Institute of Experimental Medicine, Academician Pavlov Street 12, 197376 Saint Petersburg, Russia; 5 Department of Biotechnology, I.M. Sechenov First Moscow State Medical University, Trubetskaya Street, 8–2, 119991 Moscow, Russia

**Keywords:** antibiotics, antimicrobial peptides, arenicins, complement system

## Abstract

The widespread resistance to antibiotics in pathogenic bacteria makes the development of a new generation of antimicrobials an urgent task. The development of new antibiotics must be accompanied by a comprehensive study of all of their biological activities in order to avoid adverse side-effects from their application. Some promising antibiotic prototypes derived from the structures of arenicins, antimicrobial peptides from the lugworm *Arenicola marina*, have been developed. Previously, we described the ability of natural arenicins -1 and -2 to modulate the human complement system activation in vitro. In this regard, it seems important to evaluate the effect of therapeutically promising arenicin analogues on complement activation. Here, we describe the complement-modulating activity of three such analogues, Ar-1[V8R], ALP1, and AA139. We found that the mode of action of Ar-1[V8R] and ALP1 on the complement was similar to that of natural arenicins, which can both activate and inhibit the complement, depending on the concentration. However, Ar-1[V8R] behaved predominantly as an inhibitor, showing only a moderate increase in C3a production in the alternative pathway model and no enhancement at all of the classical pathway of complement activation. In contrast, the action of ALP1 was characterized by a marked increase in the complement activation through the classical pathway in the concentration range of 2.5–20 μg/mL. At the same time, at higher concentrations (80–160 μg/mL), this peptide exhibited a complement inhibitory effect characteristic of the other arenicins. Peptide AA139, like other arenicins, exhibited an inhibitory effect on complement at a concentration of 160 μg/mL, but was much less pronounced. Overall, our results suggest that the effect on the complement system should be taken into account in the development of antibiotics based on arenicins.

## 1. Introduction

**Figure 1:**
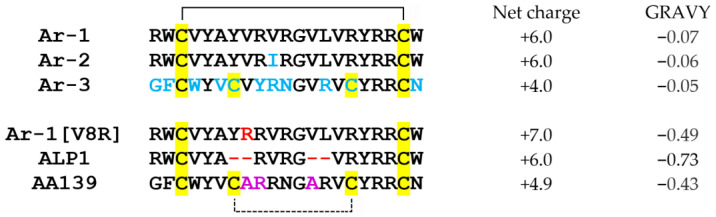
Amino acid sequences of the three natural arenicins and three analogues studied in this work. The color coding is as follows: blue, differences in primary structure of natural arenicins, taking Ar-1 as the reference; red, amino acid substitutions or deletions (indicated by the “–” symbol) in Ar-1[V8R] and ALP1 compared to Ar-1; purple, amino acid substitutions in AA139 compared to Ar-3. Cysteine residues are highlighted in yellow. Two invariant cysteines are involved in the disulfide bond common to all arenicin peptides and are shown by the solid black line in the upper part; the disulfide bond shared only by Ar-3 and its analogue, AA139, is shown by the dashed line in the bottom. All peptides have an unmodified amine and carboxyl at the N- and C-terminus, respectively. Some physicochemical properties of the peptides (net charge at pH 7.0 and hydrophobicity as the GRAVY index) are shown on the right.

Nowadays, the problem of resistance development of pathogenic microorganisms to conventional antibiotics has become increasingly serious [1]. In light of this problem, the task of searching for prototypes of a new generation of antibiotics is urgent for modern medicine. Natural antimicrobial peptides (AMPs) can be a promising source of novel antibiotics [2,3]. AMPs are short, predominantly cationic polypeptide molecules that possess toxic activity against bacteria and other pathogens. They have been described in virtually all forms of life and exhibit a great structural diversity, allowing for the most effective peptides derived from bacteria, plants, fungi, invertebrates, and vertebrates including humans to be selected as templates for antibiotic development [4]. Marine organisms were found to be an important source of AMPs [5,6,7]. In particular, arenicins from the lugworm *Arenicola marina* have attracted much attention [8]. Structurally, arenicins belong to the group of β-hairpin peptides, which are characterized by high therapeutic potential [9,10]. There are three isoforms in total: arenicin-1, -2 [11], and -3 [12] (Ar-1, -2, -3). These cationic peptides consist of 21 amino acid residues and contain one (Ar-1 and -2) or two (Ar-3) intramolecular disulfide bonds. Ar-1 and Ar-2 differ in the single amino acid residue, while the sequence of Ar-3 is more different (Figure 1).

One of the limitations of the application of AMPs as antibiotics is the possible side effects. The biological activity of AMPs is not limited to their action on microorganisms; these peptides can exhibit cytotoxic activity against host cells, display a variety of immunomodulatory effects, and participate in the pathogenesis of various diseases, for example, autoimmune diseases [13,14,15,16]. Since AMPs are considered as prototypes of a new generation of antibiotics, a comprehensive study of the possible consequences of their introduction into the human body including their immunomodulatory effects is necessary. Among the immunomodulatory effects of AMPs, one should take into account their influence on the activation of the complement system.

Complement is part of the innate immune system, which can be activated by one of three pathways: the classical pathway (CP), the alternative pathway (AP), and the lectin pathway. Complement contributes to defense against infection by opsonizing microbes with C3b, C4b, and their derivatives; by production of anaphylatoxins, C3a and C5a, which attract and activate phagocytes; and by the direct lysis of Gram-negative bacteria by the membrane attack complex (C5b-9) [17,18,19].

Previously, we found that Ar-1 and -2 as well as Ar-1-(C/A), an arenicin-1 analogue devoid of a disulfide bond due to cysteine substitutions with alanine, are able to modulate complement activation [20,21]. All three peptides affect both the classical and alternative pathways (CP and AP) of complement activation. The aim of this work was to evaluate the effect of three arenicin analogues (Figure 1), which were designed to improve the properties important for their potential as antibiotic drug progenitors.

The Ar-1[V8R] peptide differs from Ar-1 by only one amino acid residue (arginine instead of valine in position 8), but compared to natural arenicins, it exhibits a low dimerization ability in the lipid environment, which seems to largely determine the cytotoxic properties of arenicins [22,23]. The ALP1 peptide is a shortened Ar-1 analogue with reduced hydrophobicity compared to natural peptides [24]. The AA139 peptide has been developed by Adenium Biotech based on the Ar-3 sequence with three amino acid substitutions, making the peptide less hydrophobic and more positively charged [25]. The AA139 peptide is currently in preclinical development.

All of these three arenicin analogues showed an increased selectivity of action (high antibacterial and low cytotoxic activity) compared to their natural prototypes. In particular, Ar-1[V8R] exhibited bactericidal activity comparable or even slightly higher than that of natural Ar-1, but an order of magnitude higher concentrations of Ar-1[V8R] were required for similar levels of human erythrocyte lysis and cytotoxic action on human embryonic fibroblasts compared to Ar-1 [23]. ALP1 showed approximately a twofold increase in the antibacterial activity compared to Ar-1 with negligible cytotoxic activity toward various human cells (erythrocytes, embryonic fibroblasts, normal astrocytes) [24]. AA139 was non-toxic to four human cell lines, although Ar-3 showed a weak cytotoxic activity against three of them. For equal hemolytic activity toward human erythrocytes, an order of magnitude higher concentration of AA139 was required compared with Ar-3. However, in terms of antimicrobial activity, AA139 was several times more effective than Ar-3 [25,26]. Such differences in the activity pattern can be explained by the greater selectivity of AA139 to highly negatively-charged membranes characteristic of bacterial cells [26]. The AA139 peptide is also highly resistant to the inhibitory action of blood serum, in contrast to Ar-3 [25].

In this work, we investigated the immunomodulatory effects at the level of the complement system of these three arenicin analogues. by using recombinant peptides. We demonstrated that these arenicin analogues are able to affect the complement system activation in vitro.

## 2. Results

In this study, the peptides ALP1, Ar-1[V8R], and AA139 were produced as a part of the fusion proteins that included an octahistidine tag and modified thioredoxin A (M37L). The proteins were expressed in *Escherichia* *coli* BL21 (DE3) cells, and the obtained total cell lysates were fractionated by affinity chromatography. After the purification and cleavage of the fusion proteins, reverse-phase high performance liquid chromatography (RP-HPLC) was used to isolate mature AMPs. MALDI mass spectrometry analysis of recombinant AA139 showed that the measured m/z value matched that of the calculated molecular mass of the corresponding peptide, indicating the formation of two disulfide bonds and the absence of any other modifications (Figure S1). More evidence that all of the cysteine residues are involved in disulfide bridging was obtained from the alkylation experiment (Figure S2). After the repurification step (Figure S3), the final yield of the AA139 was about 4 mg per 1 L of the culture, which is comparable to that of ALP1 or Ar-1[V8R], as described previously [22,24].

**Figure 2:**
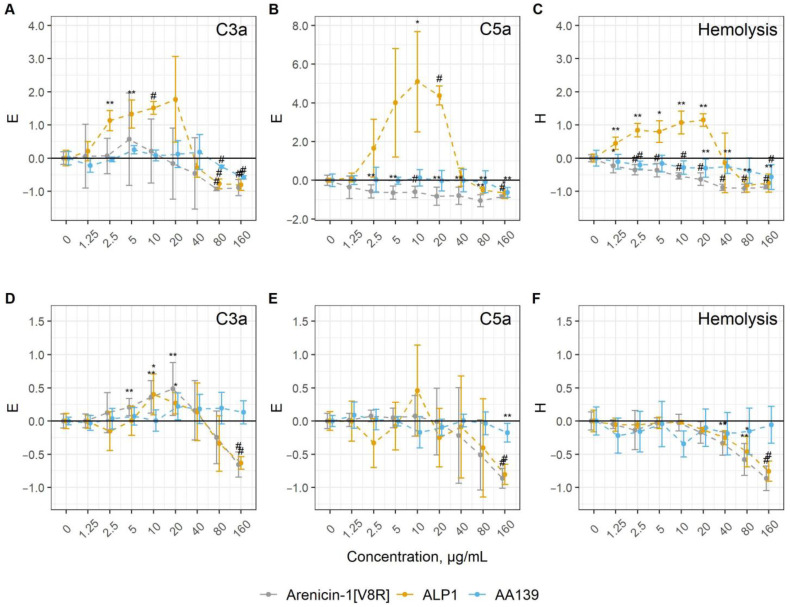
The action of arenicin analogues on complement activation, expressed in *H* and *E* coefficients. Data are represented as the mean ± standard deviation. * *p* < 0.05; ** *p* < 0.01; # *p* < 0.001 (sample vs. control). C3a accumulation (**A**,**D**), C5a accumulation (**B**,**E**) and alterations in hemolysis level (**C**,**F)** in the CP model (**A**–**C**) and AP model (**D**–**F**). See text for more information.

To evaluate the ability of arenicin peptides to modulate the human complement system, we utilized two experimental in vitro models with animal erythrocytes as targets of complement activation. Antibody-sensitized sheep erythrocytes (E^sh^) and rabbit erythrocytes (Er^rab^) were used to study the CP and AP activation in the normal human serum (NHS), respectively. In both cases, complement activation was estimated by the hemolysis level and by C3a and C5a anaphylatoxin accumulation. The results were expressed in values of *H* and *E* coefficients (Figure 2). Importantly, none of the peptides themselves led to hemolysis in experimental models since the lysis level did not differ from the baseline when NHS was replaced by the heat-inactivated serum (Table S1).

All of the peptides demonstrated the ability to modulate complement activation albeit in different modes. In the presence of antibody sensitized sheep erythrocytes E^sh^ (the CP model), the dose-dependent action of the peptides on C3a accumulation was observed. However, Ar-1[V8R] and AA139 only led to the inhibition of C3a production at concentrations of 80 and 160 μg/mL (*E* coefficient values below zero), whereas ALP1 displayed a bidirectional effect. The addition of the latter peptide at 2.5–20 μg/mL resulted in the elevated C3a level in the experimental samples, but at higher concentrations of ALP1, the C3a level was decreased compared to the control without peptides (Figure 2A). Similar patterns persisted in the analysis of C5a accumulation and hemolysis level (Figure 2B,C). Of note is the extremely high complement activation in the presence of ALP1, with a six-fold increase in the C5a level (corresponding to an *E* coefficient value of 5).

In the AP model, the AA139 peptide had no apparent effect on the complement activation, only the C5a level at a peptide concentration of 160 µg/mL was significantly lower than that of the control. The two Ar-1 analogues mainly showed an inhibitory effect at high concentrations (160 µg/mL for the assessment of the C3a and C5a levels, or starting from 40 µg/mL for the level of hemolysis). At the same time, increased C3a levels were observed in the presence of Ar-1[V8R] and ALP1 at 5–20 µg/mL, which were not reflected in the C5a levels or Er^rab^ lysis (Figure 2E,F).

Quantification of the inhibitory effect of arenicin analogues on complement activation, presented as IC_50_ values (concentrations corresponding the *E* or *H* value of −0.5), is shown in Table 1.

The data in Table 1 show that Ar-1[V8R] was the most efficient complement inhibitor among these three peptides as it had the lowest IC_50_ values (in μM), whereas AA139 was only a weak CP inhibitor.

## 3. Discussion

The development of new therapeutic drugs including antibiotics should be accompanied by a thorough investigation of all aspects of their possible effects in the body, in particular, their action on complement activation. Although the complement system, as part of the immune system, contributes to the microbe clearance, its excessive activation is generally undesirable. As a complex multifaceted system, complement performs a variety of immune and non-immune functions and may be involved in the development of many pathological processes if it is dysregulated. In this regard, the therapeutic inhibition of complement, rather than its stimulation, is currently more urgent in medical practice [27,28,29]. Thus, the property of a drug candidate to enhance complement activation can be regarded as an unfavorable side effect. In particular, this refers to the increased production of proinflammatory factors such as C3a and C5a. On the other hand, the inhibition of complement by an antibiotic may impair the antimicrobial response of the immune system and thus reduce the efficacy of the antibiotic in vivo. Nevertheless, the anti-inflammatory and immunosuppressive activity of antibiotics is beneficial in conditions of excessive inflammation [30,31]. Therefore, the ability of a drug candidate to enhance or inhibit complement activation needs to be taken into account when developing therapeutic protocols. It should be noted that the concentration-dependent bidirectional effect of the drug candidate on complement is a critical issue, as it can lead to unpredictable effects in vivo.

In previous works, we found that Ar-1 and Ar-2 are able to modulate both the CP and AP of the complement, leading to the enhancement or inhibition of activation depending on their concentrations [20,21]. It has also been shown that Ar-1 is able to interact with two complement proteins, C1q and C3b, which may explain its action on the two activation pathways [32,33]. Despite the generally similar mode of action on complement activation and the difference in a single amino acid residue, some details of the effects of Ar-1 and Ar-2 differed markedly. In particular, the ability to enhance complement CP activation at relatively low concentrations is much weaker for Ar-2 than for Ar-1 [21].

Arenicins, as biologically active peptides, have attracted the attention of researchers. A number of works are devoted to obtaining modified analogues with altered functional activities [21,34,35,36]. Three previously described arenicin analogues, Ar-1[V8R] [22], ALP1 [24], and AA139 [25], became the subject of study in the present work. Their high selectivity makes these peptides promising prototypes of new antibiotics. In particular, comprehensive preclinical studies are being conducted with AA139 peptide, which has shown good effectiveness against Gram-negative bacteria including antibiotic-resistant strains both in vitro and in animal models [25,26,37,38].

In our work, we investigated the effect of these three analogues on the activation of the human complement system in vitro. We used recombinant peptides for the experiments. Two rounds of HPLC purification assured the absence of bacterial contaminants.

We showed that the action of Ar-1[V8R] and ALP1 was similar to that of Ar-1 and Ar-2, as previously described. However, although both peptides are derivatives of Ar-1, Ar-1[V8R] is more similar to Ar-2 in its action. Moreover, of five of the highly similar arenicin isoforms we studied (Ar-1, Ar-2, and their analogues), Ar-1[V8R] was the only one whose enhancing effect on complement CP activation was negligible. If the weak enhancement of C3a production in the AP model is not taken into account, this peptide can be called a pure complement inhibitor. Interestingly, of all the arenicin peptides studied thus far, Ar-1[V8R] is the most effective in terms of complement inhibition. In hemolytic assays, its IC_50_ values are 3.2 and 23.8 μM for CP and AP, respectively. In contrast, ALP1 was the strongest enhancer of the CP complement activation of all of the natural isoforms and their analogues studied. At the same time, at high concentrations (80–160 µg/mL), this peptide exhibited the complement inhibitory effect characteristic of the other arenicins. The inhibitory action of the AA139 peptide on complement was much weaker compared to other arenicins. It is difficult to say whether these results for AA139 are due to sequence differences between Ar-3 and other arenicins or with structural features of this particular analogue.

The mechanisms of modulation of the complement system activation by arenicins remain unclear, especially the reasons for the bidirectional action of some isoforms. Apparently, this is at least partly due to the interaction of arenicins with complement proteins (C1q, C3b), but other mechanisms are also possible, for example, heparin binding, as discussed in [21]. The reasons for the differences in the action of the different arenicin isoforms are also elusive, but they seem to be more related to specific amino acid residues or sequences rather than to differences in the physicochemical properties. In this regard, it can be noted that of the six arenicin peptides shown in Figure 1, the least hydrophobic (AA139) and one of the most hydrophobic (Ar-1) peptides exhibited the greatest ability to enhance complement activation. It is possible, however, that the peptide size may be important. Thus, ALP-1 was designed to mimic tachyplesins, AMPs from horseshoe crabs *Tachypleus* spp., with the same polypeptide chain length. As we describe here for ALP-1, tachyplesin-1 was shown to be an enhancer of complement activation [39]. Further studies are needed to understand which structural features of arenicins determine their action on complement activation and, consequently, how they can be modified to alter these properties in a targeted manner.

In terms of the potential applications of the studied arenicin analogues as therapeutic antibiotics, ALP1 needs further modification to remove its bidirectional effect on complement, and first, its ability to significantly enhance the production of C5a anaphylatoxin, which has potent proinflammatory activity. It seems that for Ar-1[V8R], and especially for AA139, their possible effects on complement activation should not be a significant obstacle to their therapeutic use. However, high local concentrations of the peptides in the bloodstream should be avoided in order to prevent undesirable complement inhibition.

It should be noted that our results were obtained in experiments in vitro and it is possible that they will not be fully reproducible in vivo, and, therefore, the recommendations given are tentative. Particular details of the experimental conditions (use of diluted serum, presence of gelatin in the buffers, etc.) can affect the interaction of peptides with complement proteins. One reason to be cautious about extrapolating the in vitro results to the in vivo conditions is the presence of proteinases in the serum, which can reduce the stability of the peptides. In our work, we incubated the peptides with serum for 30 min, which allows us to consider such risks as minimal. These considerations imply the need for further investigations including in clinical trials.

## 4. Materials and Methods

### 4.1. Peptides

The recombinant Ar-1[V8R] and ALP1 were obtained as described previously [22,24]. The peptide AA139 was obtained using the same procedures. Briefly, the gene encoding AA139 was obtained by the annealing of two primers followed by one-round of DNA-polymerase extension and then cloned into the pET-based vector as described previously [22,24]. The target peptides were expressed in *E. coli* BL21 (DE3) as chimeric proteins that included the octahistidine tag, the *E. coli* thioredoxin A with the M37L substitution (TrxL), methionine residue, and a mature peptide. The transformed cells were grown at 37 °C in Lysogeny broth (LB) medium supplemented with 20 mM glucose, 1 mM magnesium sulfate, and 100 μg/mL ampicillin were induced at OD_600_ 0.8 with 0.3 mM isopropyl β-D-1-thiogalactopyranoside (IPTG) for 4 h at 30 °C and 220 rpm. After centrifugation, the pelleted cells were suspended and sonicated in the 100 mM phosphate buffer (pH 7.8) containing 20 mM imidazole and 6 M guanidine hydrochloride to fully solubilize the fusion protein. Purification of the peptide involved immobilized metal affinity chromatography (IMAC) of the cell lysate, CNBr cleavage of the fusion protein, and reversed-phase HPLC (RP-HPLC) with the use of a Reprosil-pur C_18_-AQ column (Dr. Maisch GmbH). The collected fractions were analyzed by MALDI-TOF mass-spectrometry using a Reflex III instrument (Bruker Daltonics). The fractions containing the target peptides were lyophilized and dissolved in water. The peptide concentrations were estimated using UV absorbance. The fractions with confirmed masses were dried in vacuo and repurified by the second round of RP-HPLC. Repurification of peptides was performed using the analytical column (Symmetry 300 C_18_) at a flow rate of 1 mL/min in a linear gradient of solution B (80% acetonitrile, 0.1% TFA) in solution A (5% acetonitrile, 0.1% TFA): 0–100% for 50 min (Figure S3).

The intramolecular disulfide bonds formation in AA139 was confirmed using alkylation with iodoacetamide (IAA). Two peptide aliquots (80 μM in 95 μL of 100 мM potassium phosphate buffer, pH 7.8), one of which was supplemented with 2 mM dithiothreitol (DTT), were incubated at 55 °C for 30 min. Then, 5 μL of a freshly prepared 400 mM aqueous IAA solution were added to both tubes, and the samples were incubated at room temperature in the dark for another 30 min. The samples were then desalted using ZipTip-C_18_ pipette tips (Merck-Millipore) and analyzed by MALDI-TOF mass-spectrometry.

### 4.2. Serum and Erythrocytes

Normal human serum (NHS) used as a source of complement was collected by medical staff (Laboratory of Viral Infections Diagnostics, Department of Clinical Microbiology, Pavlov First Saint Petersburg State Medical University, Saint Petersburg, Russia) from more than 20 healthy volunteers, pooled, aliquoted, and stored at −70 °C no longer than two months. Serum aliquots were thawed at +4 °C on the day of the experiment, kept in an ice bath before introducing to test tubes, and were not used repetitively. To obtain serum with inactivated complement, it was incubated at +56 °C for an hour immediately before the experiment.

Animal erythrocytes were purified from whole blood of rabbit and sheep and stored in Alsever’s solution at +4 °C. Before use, they were washed with appropriate buffer: DGVB^++^ (dextrose gelatin veronal buffer with Ca^2+^ and Mg^2+^) for sheep erythrocytes (E^sh^) and GVB^+^ (gelatin veronal buffer with Mg^2+^) for rabbit erythrocytes (E^rab^). DGVB^++^ is 4.5 mM sodium barbital buffer containing 150 mM NaCl, 15 mM glucose, 0.15 mM CaCl_2_, 1 mM MgCl_2_, 0.05% gelatin; pH 7.4. GVB^+^ is 4.5 mM sodium barbital buffer containing 150 mM NaCl, 10 mM Mg-EGTA, 0.05% gelatin; pH 7.4. Before the experiments, E^sh^ was sensitized with antibodies (anti-sheep red blood cell stroma antibodies produced in rabbits, S1389, Sigma, St. Louis, MO, USA) diluted 1:1600 for 40 min at +37 °C.

### 4.3. Complement Activation

The ability of peptides to modulate the human complement system was evaluated by hemolytic assay and by ELISA, as previously described [20,21]. In addition to C3a ELISA, as in previous works, we used the C5a ELISA Kit from “Cytokine” (Saint Petersburg, Russia).

Briefly, the experimental samples contained erythrocytes, diluted NHS as a source of complement proteins, and arenicin analogues at different concentrations. For the CP assay, E^sh^ was introduced to a final concentration of 1 × 10^8^ cells per mL, NHS was diluted to 1%, and DGVB^++^ was used to dilute all of the components. For the AP assay, there were 1 × 10^8^ cells per mL of E^rab^, 5% NHS, and GVB^+^. After the 30 min incubation at +37 °C, the lysis of the erythrocytes was stopped by the addition of PBS (phosphate buffered saline, pH 7.4) in a ratio of 1:7.5. Samples were centrifuged at 500 g for 5 min at room temperature and the supernatants were photometered at 414 nm. The same supernatants were used for C3a and C5a determination by ELISA.

For the calculation and visualization of results, we utilized the coefficients for the evaluation of the hemolytic activity of complement (*H*, for “hemolysis”) and of complement-dependent C3a accumulation (*E*, for “ELISA”).

The hemolytic activity of serum in a sample was counted as

$$H=\frac{\mathrm{OD}414(\mathrm{sample})-\mathrm{OD}414(\mathrm{control})}{\mathrm{OD}414(\mathrm{control})}$$

The control was a sample with no peptides added. The *H* values above zero indicate the augmentation of complement-mediated hemolysis, while the *H* values below zero mean inhibition.

The alterations in the accumulation of C3a or C5a were expressed as

$$E=\frac{\mathrm{OD}450(\mathrm{sample})-\mathrm{OD}450(\mathrm{control})}{\mathrm{OD}450(\mathrm{control})}$$

As the control, a sample with no peptides added was used. As with the *H* coefficient, the *E* values above or below zero indicate an increase or decrease in anaphylatoxin accumulation, respectively.

### 4.4. Statistical Analysis

Statistical analysis was made using R language (v4.0.2) in RStudio environment (R Core Team, R Foundation for Statistical Computing, Vienna, Austria). Significance of the *H* and *E* coefficient values’ deviation from the controls was evaluated by the two-sample *t*-test. The experiments on complement modulation were performed at least four times for each of the peptides. For both the hemolytic and ELISA assays, *p*-values less than 0.05 were considered statistically significant. The plots were drawn using R language with the ggplot2 (v3.3.2) and ggpubr (v.0.4.0) packages.

## Figures and Tables

**Table 1:** IC_50_ values, μg/mL (μM). “-”—50% inhibition is not achieved in the concentration range of 0–160 μg/mL.

Peptide	Classical Pathway Model			Alternative Pathway Model		
	C3a	C5a	Hemolysis	C3a	C5a	Hemolysis
Ar-1[V8R]	43.4 (15.4)	2.1 (0.75)	8.9 (3.2)	129.6 (46.0) *	78.7 (28.0)	67.0 (23.8)
ALP1	57.3 (25.1) *	79.2 (34.7) *	61.4 (26.9) *	123.7 (54.2) *	98.8 (43.3)	90.5 (39.6)
AA139	140.2 (55.0)	141.4 (55.5)	130.6 (51.2)	-	-	-

* Bidirectional action was observed with signal enhancement at lower concentrations.

## Data Availability

All data presented in this study are available from the corresponding author on reasonable request.

## Supplementary Materials

The following supporting information can be downloaded at: https://www.mdpi.com/article/10.3390/md20100612/s1, Figure S1: MALDI-MS analysis of the recombinant AA139; Figure S2: MALDI-MS analysis of the alkylated AA139; Figure S3: Repurification of the recombinant AA139; Table S1: Evaluation of the hemolytic activity of arenicin analogues in heat-inactivated serum.
