# Supplementary material for: In Vitro Modulation of Complement Activation by Therapeutically Prospective Analogues of the Marine Polychaeta Arenicin Peptides
Source: Mar Drugs. 2022 Sep 28;20(10):612. doi: 10.3390/md20100612 (PMC9605166; doi:10.3390/md20100612)
Supplement: Supplementary file 1 [file marinedrugs-20-00612-s001.zip › marinedrugs-1900558-supplementary.pdf]

Supplementary Information

In vitro modulation of complement activation by therapeutically prospective analogues of the marine polychaeta arenicin peptides

Ilia A. Krenev [1], Pavel V. Panteleev [2], Ekaterina S. Umnyakova [1,3], Nikolay P. Gorbunov [4], Valeria A. Kostevich [4], Sergey V. Balandin [2], Tatiana V. Ovchinnikova [2,5], Galina M. Aleshina [1] and Mikhail N. Berlov [1,*]

[1] Department of General Pathology and Pathological Physiology, Institute of Experimental Medicine, Acad. Pavlov Str. 12, 197376 Saint Petersburg, Russia

[2] M.M. Shemyakin and Yu. A. Ovchinnikov Institute of Bioorganic Chemistry, Russian Academy of Sciences, Miklukho-Maklaya Str., 16/10, 117997 Moscow, Russia

[3] Department of Pharmaceutical Sciences, University of Basel, Klingelbergstrasse 50, 4056 Basel, Switzerland

[4] Department of Molecular Genetics, Institute of Experimental Medicine, Acad. Pavlov Str. 12, 197376 Saint Petersburg, Russia

[5] Department of Biotechnology, I.M. Sechenov First Moscow State Medical University, Trubetskaya Str., 8-2, 119991 Moscow, Russia

* Correspondence: berlov.mn@iemspb.ru; Tel.: +7-911-936-9839

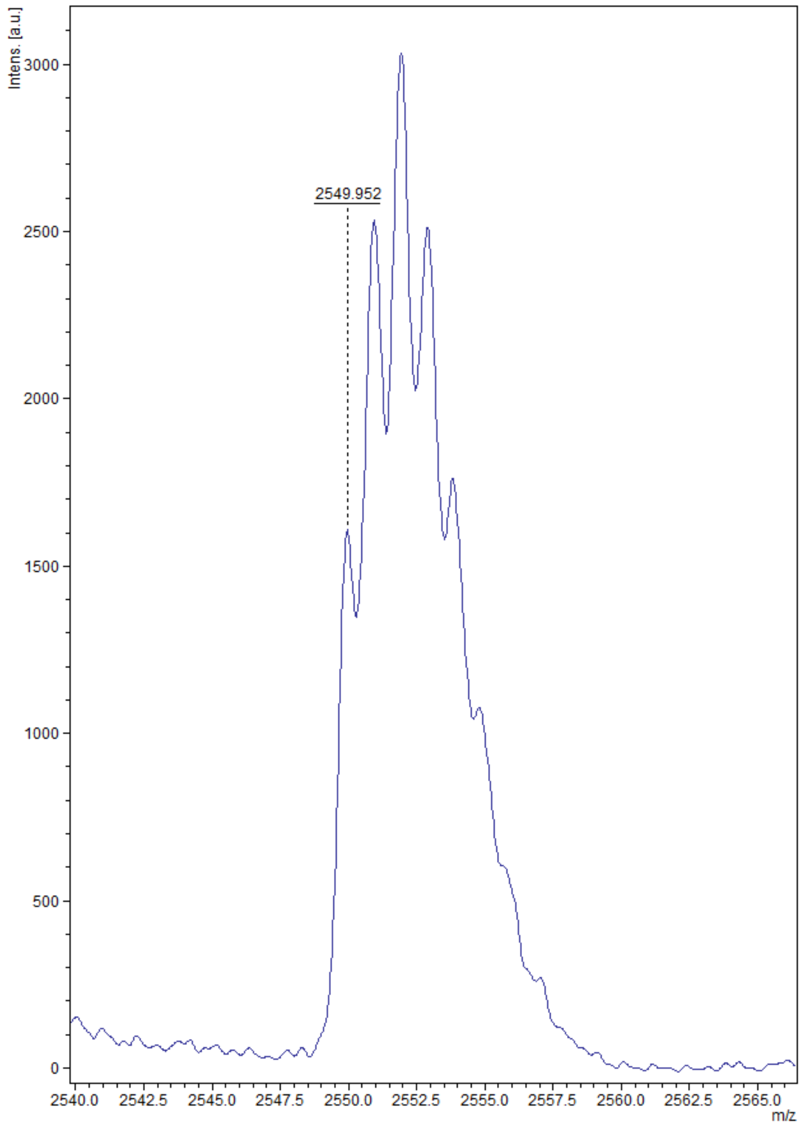

Figure S1. MALDI-MS analysis of the recombinant AA139. Calculated [M+H]+ monoisotopic molecular mass of AA139 is of 2549.17.

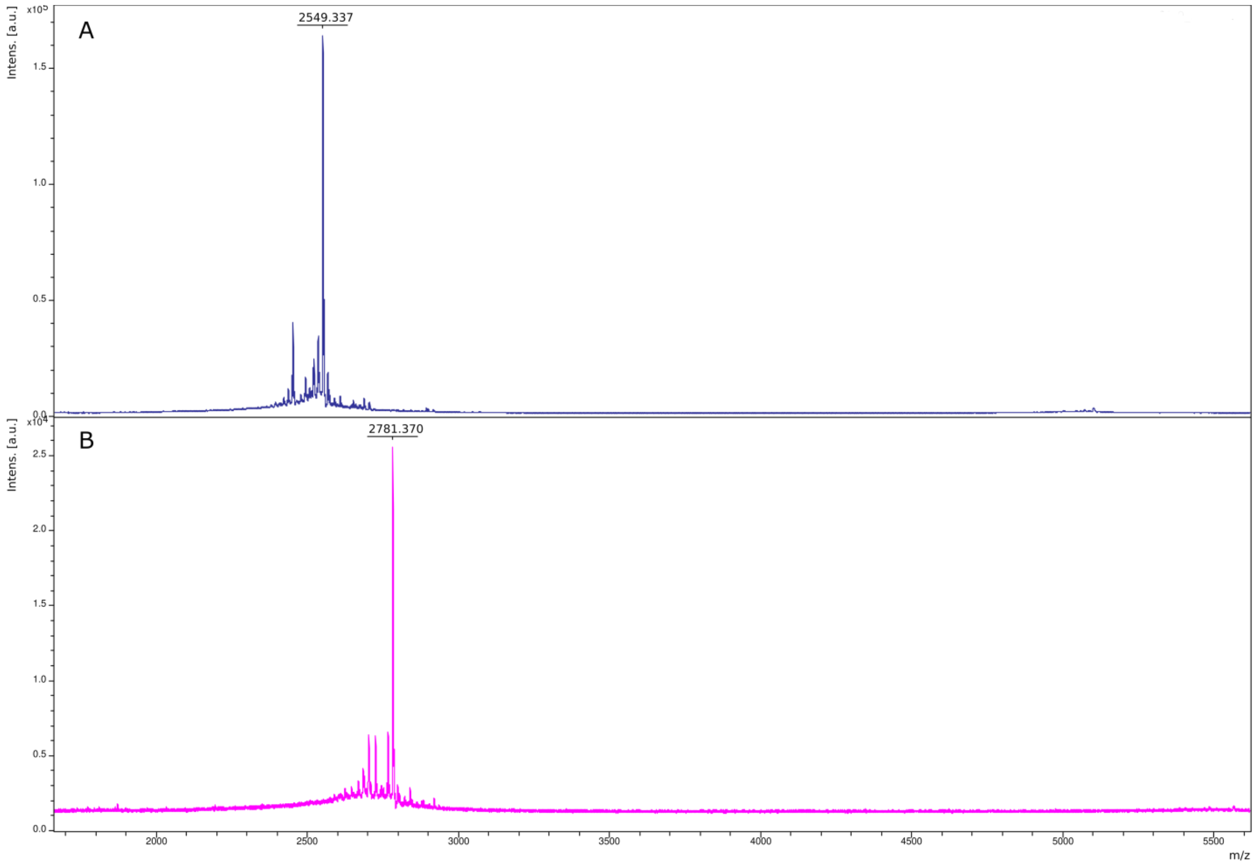

Figure S2. MALDI-MS analysis of the recombinant AA139 after incubation with iodoacetamide (IAA) w/o (A) or after (B) the addition of DTT. The difference between the two m/z values (232 Da) corresponds to 4 acetamide residues which indicates the absence of free thiol groups in the recombinant peptide.

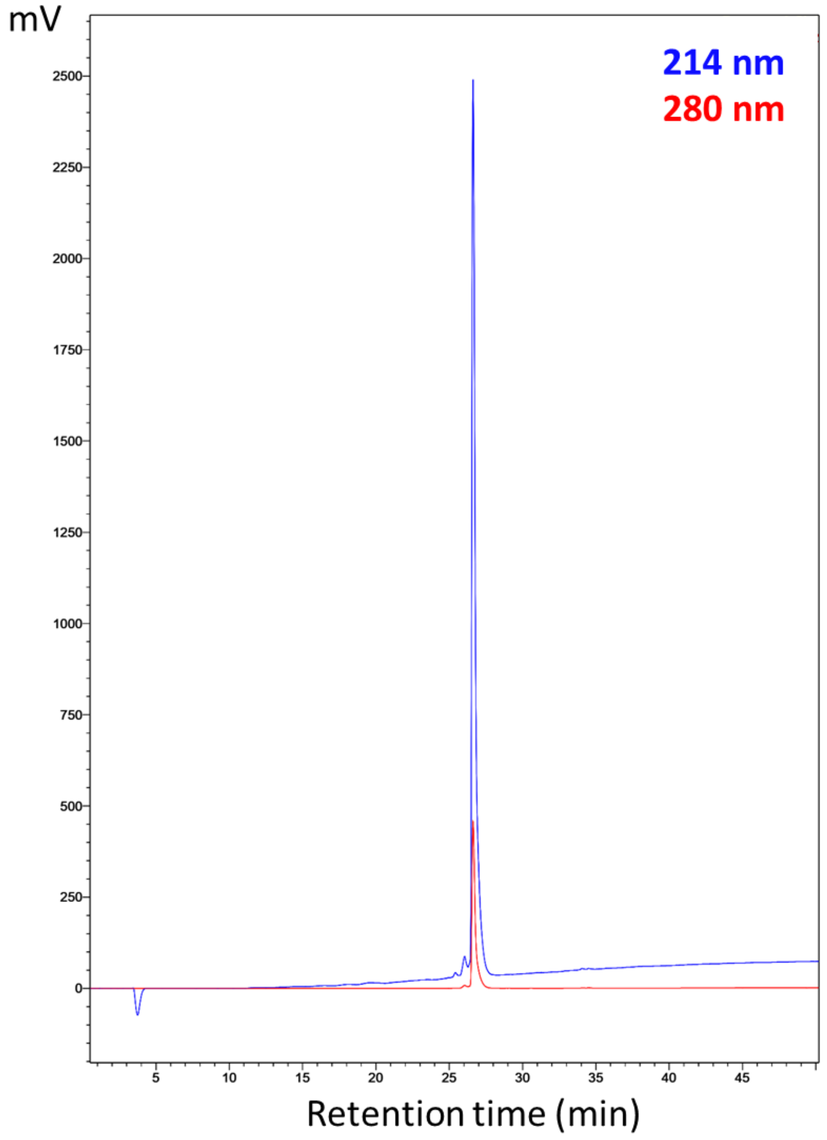

Figure S3. Repurification of the recombinant AA139. RP-HPLC was performed using the analytical column (Symmetry 300 C18) at a flow rate of 1 mL/min in a linear gradient of solution B (80% acetonitrile, 0.1% TFA) in solution A (5% acetonitrile, 0.1% TFA): 0-100% for 50 min.

Table S1. Evaluation of the hemolytic activity of arenicin analogues in heat-inactivated serum. Peptides at 160 µg/mL were incubated with Er^{sh} or Er^{rab} in presence of heat-inactivated serum at +37°C for 30 minutes, then the samples were diluted with PBS as described in section 4.3. For 100% lysis, distilled H_2O was added to the samples instead of PBS. The average values from the two experiments are shown.

	OD_{414}	
	Er^{sh}	Er^{rab}
100% lysis	0.995	0.878
Without peptide	0.006	0.002
Ar-1[V8R]	0.003	0.002
ALP1	0.005	0.004
AA139	0.003	0.003
